# Available processing resources influence encoding-related brain activity before an event

**DOI:** 10.1016/j.cortex.2012.10.011

**Published:** 2013-09

**Authors:** Giulia Galli, A. Dorothea Gebert, Leun J. Otten

**Affiliations:** Institute of Cognitive Neuroscience, University College London (UCL), London, UK

**Keywords:** Prestimulus brain activity, Long-term memory, Encoding, Processing resources, Divided attention

## Abstract

Effective cognitive functioning not only relies on brain activity elicited by an event, but also on activity that precedes it. This has been demonstrated in a number of cognitive domains, including memory. Here, we show that brain activity that precedes the effective encoding of a word into long-term memory depends on the availability of sufficient processing resources. We recorded electrical brain activity from the scalps of healthy adult men and women while they memorized intermixed visual and auditory words for later recall. Each word was preceded by a cue that indicated the modality of the upcoming word. The degree to which processing resources were available before word onset was manipulated by asking participants to make an easy or difficult perceptual discrimination on the cue. Brain activity before word onset predicted later recall of the word, but only in the easy discrimination condition. These findings indicate that anticipatory influences on long-term memory are limited in capacity and sensitive to the degree to which attention is divided between tasks. Prestimulus activity that affects later encoding can only be engaged when the necessary cognitive resources can be allocated to the encoding process.

## 1 Introduction

The pattern of brain activity that precedes an event can influence the way the event is processed. It has been shown that activity within a few seconds of an imminent event can indicate how that event will be perceived, attended, emotionally processed, decided upon, and acted upon (e.g., Cunnington et al., 2003; Driver and Frith, 2000; Hesselmann et al., 2008; Mackiewicz et al., 2006; Shibata et al., 2008). In the area of long-term memory, prestimulus activity contributes to the likelihood that retrieval will be successful. Activity before event onset may reflect a state that encourages events to be treated as retrieval cues and orient the search through memory toward relevant kinds of information (Rugg and Wilding, 2000).

More recently, prestimulus activity has been shown to also affect the initial encoding of an event into long-term memory. There are now a good number of studies that have demonstrated that brain activity elicited by a cue that gives advance information about an upcoming event can predict whether that event will be remembered or forgotten in a later memory test. This activity is therefore thought to play a role in effective encoding (Paller and Wagner, 2002). Encoding-related activity before an event has been shown using functional magnetic resonance imaging (Adcock et al., 2006; Bollinger et al., 2010; Mackiewicz et al., 2006; Park and Rugg, 2010; Uncapher et al., 2011; Wittmann et al., 2005, 2007), magnetoencephalography (Düzel et al., 2005; Guderian et al., 2009), scalp-recorded electroencephalography (Galli et al., 2011, 2012; Gruber and Otten, 2010; Otten et al., 2006, 2010; Padovani et al., 2011) and intracranial recordings (Fell et al., 2011; Rutishauser et al., 2010). Prestimulus activity can affect the encoding of a variety of stimulus events, especially in deep processing tasks, and is dissociable from encoding-related activity after an event (Galli et al., 2011; Otten et al., 2006, 2010). The main brain regions implicated thus far are the medial temporal lobe and midbrain (Adcock et al., 2006; Fell et al., 2011; Guderian et al., 2009; Mackiewicz et al., 2006; Park and Rugg, 2010; Rutishauser et al., 2010; Wittmann et al., 2005, 2007).

The role that prestimulus activity plays in memory encoding is unknown. Generally speaking, such activity may reflect a neural context that is conducive to encoding (Meeter et al., 2004; Yoo et al., 2012), an active preparatory process (Otten et al., 2010) or perhaps an increase in attention or arousal that strengthens later memory-related processes (Park and Rugg, 2010). To help discern its functional role, we used a dual task paradigm in the present experiment to assess how encoding-related activity varies as a function of the amount of processing resources that are available before event onset. The idea behind this paradigm is to tax the system's limited pool of resources and interfere with the encoding process by way of a secondary task. If encoding-related processes before an event are sensitive to the division of attention between tasks, such processes may be limited in capacity and not able to operate independently (Pashler, 1994). This would imply that sufficient processing resources are needed to engage encoding-related activity before event onset. If, in contrast, encoding-related processes proceed relatively automatically without being dependent on resource-availability, prestimulus activity would be expected to be similar in size regardless of the difficulty of a secondary task. Although the concept of ‘resources’ has received substantial criticism (e.g., Navon, 1984), the dual task paradigm has made a significant contribution to our understanding of the functional and neural architecture in health and disease (e.g., Bonato et al., 2010; Wild-Wall et al., 2011).

The degree to which encoding-related processes rely on processing resources has been investigated extensively for neural activity that follows an event. This work has shown that explicit memory critically depends on the deployment of processing resources. The overall amount of attention paid to an event, and which aspects of the event are attended, determine the size and type of encoding-related neural activity elicited by the event (e.g., Mangels et al., 2001; Uncapher et al., 2011). With respect to memory performance, at least a basic level of resources needs to be allocated to an event when it is first experienced for memory to be successful. Performing a secondary task while encoding an event into memory makes it less likely that the event will later be retrieved, and retrieval success furthermore varies with the emphasis that is placed on the secondary task (Craik and Lockhart, 1972; Hicks and Marsh, 2000). Such performance differences are typically interpreted as being due to encoding-related processes after event onset.

The aim of the present experiment was to assess whether encoding-related processes before event onset also depend on the degree to which processing resources are available. Engaging prestimulus activity that is relevant for encoding may compete with other ongoing processes. Two observations in the literature hint that this might be the case. First, prestimulus activity is sensitive to a match between the input modalities of the to-be-encoded event and preceding cue. Prestimulus activity affects the encoding of visual words when the cue is also visual in nature, but not when it is auditory (Otten et al., 2006, 2010). A mismatch in input modalities may necessitate an initial reorienting of attention toward the other modality, leaving insufficient resources to also set up brain activity that helps encoding. Second, a functional magnetic resonance imaging study has shown that encoding-related brain activity before a visual object differs depending on whether the object occurs in an expected or unexpected location (Uncapher et al., 2011). This has been taken to suggest that prestimulus activity is sensitive to where attention is directed. Following on from these observations, the present experiment evaluated whether encoding-related activity before event onset is affected by the degree to which processing resources are available.

We recorded electrical brain activity from the scalps of healthy adults while they memorized short lists of intermixed visual and auditory words for later free recall. A cue presented just before word onset signaled the upcoming input modality. A visual cue signaled a visual word, and an auditory cue an auditory word. The deployment of processing resources before word onset was manipulated by asking participants to perform a perceptual discrimination task on the cue as well as prepare for the upcoming memorization. The difficulty of the discrimination task was varied across task blocks by making the cues more or less similar to one another. A more difficult discrimination was presumed to require more processing resources, leaving fewer resources to also set up preparatory encoding-related activity. The question of interest was how encoding-related activity before word onset varies as a function of discrimination difficulty. If encoding-related activity primarily occurs in the context of easy cue discriminations, this would lend support to the view that the activity is limited in capacity and sensitive to available processing resources.

## 2 Method

### 2.1 Participants

The experimental procedures were approved by the University College London Research Ethics Committee. Twenty-eight volunteers [mean age = 21.5 years, standard deviation (SD) = 2.1, 10 men] were remunerated at a rate of £7.50/h for their participation. All were right-handed, had normal or corrected-to-normal vision, and reported to be native English speakers without psychiatric or neurological illnesses. All participants provided written informed consent before participating.

### 2.2 Memory task

**Fig. 1:**
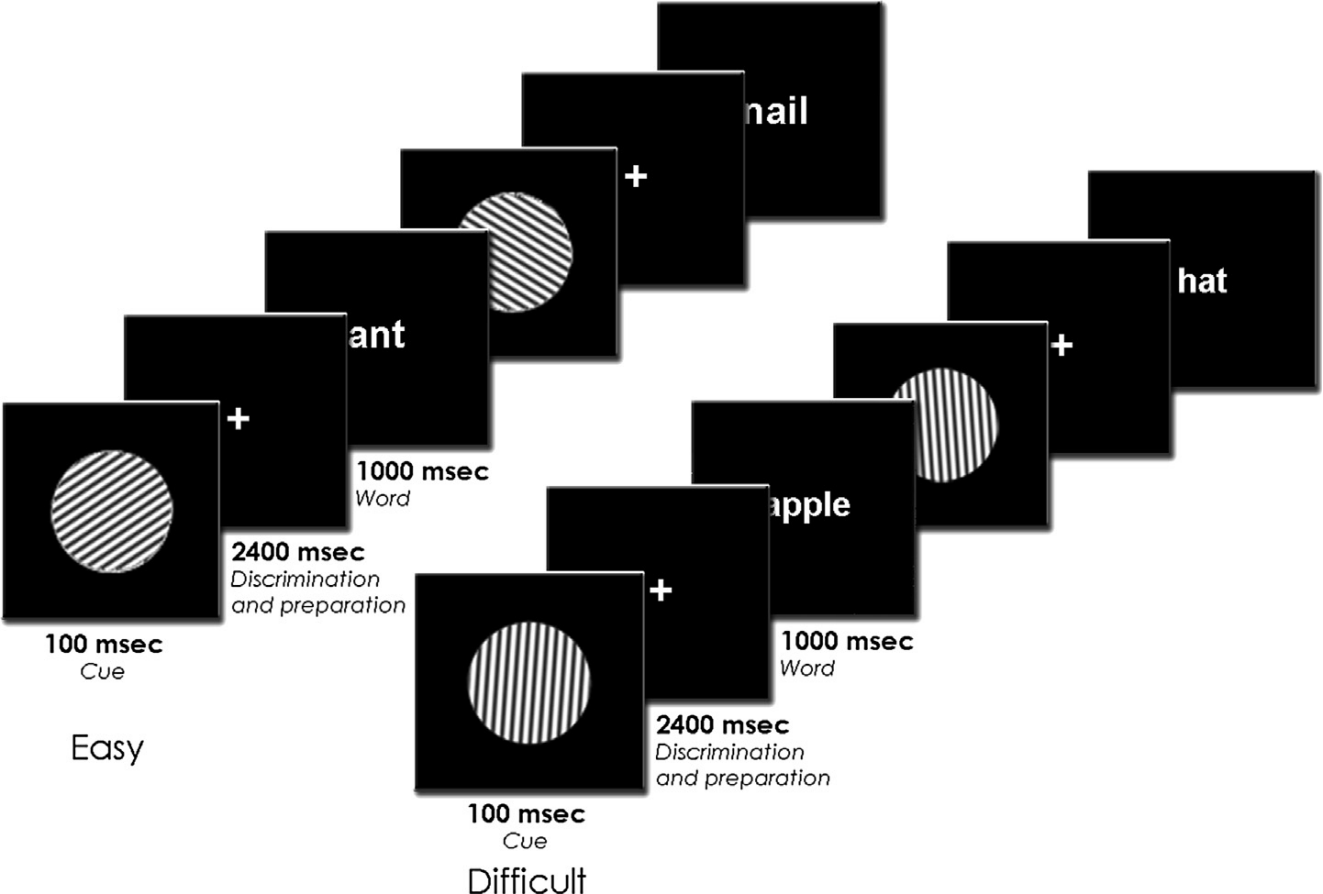
Schematic illustration of trial sequences in the easy and difficult versions of the memorization task. The example shows trials in the visual modality. Participants were asked to memorize the words for later free recall. Visual words were preceded by gratings that were either easy to discriminate (oriented 85° to the left or right) or difficult to discriminate (oriented 45° to the left or right). In both cases, one of two buttons had to be pressed according to the orientation of the cue. The experiment also included trials in the auditory modality. In these, auditory words were preceded by pure tones that differed in frequency by a large (300/2300 Hz) or small (700/1700 Hz) amount.

The experiment involved the intentional memorization of short lists of words, each followed by free recall. Participants were seated in front of a computer monitor and given a pen and clipboard with 24 blank recall sheets. They then memorized 24 lists of 16 words (concrete nouns, 3–12 letters, 0–500 occurrences/million; Kučera and Francis, 1967). Each list contained eight randomly intermixed visual words (white Helvetica font, 500 msec duration, visual angle of ∼.7° vertically and 1–4.5° horizontally) and auditory words (British adult male voice, 650 msec mean duration, range 310–1130 msec). Before the onset of each word, a cue was presented to signal the upcoming input modality (Fig. 1). Visual words were always preceded by visual cues (gratings, visual angle of 2° horizontally and vertically, four cycles/degree spatial frequency, 50% contrast) and auditory words by auditory cues (pure tones). Participants were encouraged to use the cues to prepare for the memorization of the upcoming word. Words had to be memorized using an elaborative rehearsal strategy, that is, by connecting the words in a list in a meaningful way via images or stories (cf. Galli et al., 2012). At the end of each list, a distractor task was performed for 30 sec to avoid recency effects in the free recall task. Participants counted backward in threes starting with a random number between 81 and 99 displayed on the screen. Participants were then given 1 min to write down as many words as they could remember from the preceding list. Words could be recalled in any order.

In addition to memorizing the words, participants were asked to perform a perceptual discrimination task on the prestimulus cues. This was done to manipulate the degree to which processing resources are available before word onset. For visual cues, the task consisted of judging whether the grating was oriented to the left or right. For auditory cues, the decision was whether the tone was low or high in frequency. One of two buttons had to be depressed according to a participant's decision. The left index finger was always assigned to left orientations and low tones, and the right index finger to right orientations and high tones, to maintain natural stimulus-response mappings (Rusconi et al., 2006). Participants were asked to both discriminate the cues and prepare for the upcoming memorization, with no further instructions about which task to prioritize.

The difficulty of the perceptual discrimination task was manipulated across word lists. This was done to give participants maximum opportunity to set up and maintain a consistent level of attention across trials. Randomly intermixing easy and difficult discrimination trials may encourage the two types of trial to be treated relatively similarly (e.g., Galli and Otten, 2011). A block design also avoided the interpretational problems engendered by intermixing four different visual and four different auditory cues. In the easy discrimination condition, visual cues had large differences in grating orientation (−85°/85°) and auditory cues large differences in tone frequency (300/2300 Hz). In the difficult discrimination condition, these differences were considerably smaller (−45°/45° for visual cues and 700/1700 Hz for auditory cues). Of the 24 word lists, half were memorized while performing easy cue discriminations and half while performing difficult cue discriminations. Six lists in each difficulty condition were presented consecutively, with presentation order of the blocks counterbalanced across participants. Different word lists were created such that across participants, each critical word appeared equally often in the visual and auditory modality and in the easy and difficult cue discrimination conditions. Participants practiced with two word lists, one for each discrimination condition, before starting the experimental lists.

Cues were presented for 100 msec, starting 2.5 sec before word onset. This interval is longer than the 1.5 sec employed in our previous prestimulus work with auditory and visual stimuli (Galli et al., 2012; Otten et al., 2006, 2010). Pilot work indicated that participants could not both perform the cue discrimination task and memorize the word when the cue-word interval was too short. We therefore opted for a longer interval to maintain acceptable discrimination and memory performance. The time in between successive cue onsets varied randomly between 5 and 5.5 sec. A fixation point (a plus sign) was continuously present on the screen except when words and cues were presented.

### 2.3 Simple discrimination tasks

Before memorizing the word lists, we asked participants to perform two simple perceptual discrimination tasks (hereafter referred to as Task 1 and Task 2) to help understand the findings obtained in the memorization task. These tasks also allowed participants to practice the perceptual discriminations. In Task 1, the gratings and pure tones used as cues in the memorization task were presented in isolation. Visual and auditory stimuli were randomly intermixed and separated by an interval that varied randomly between 2 and 2.5 sec. In one block of 48 trials, the stimuli associated with the easy discrimination were presented (gratings tilted 85° to the left or right and 300 or 2300 Hz tones). In another block of 48 trials, the more subtle differences had to be discriminated (gratings tilted 45° and 700/1700 Hz tones). The decisions and response assignments were identical to those used for cue discriminations in the memorization task. In Task 2, the same stimulus sequence was employed as in the memorization task except that neutral stimuli rather than words were presented. This was done to assess perceptual discriminations in the context of interspersed stimulus events. The visual and auditory cues were the same as those used before, but this time they were presented 2.5 sec before the string “xxxxxx” or the sound corresponding to the letter “x”, respectively. The time in between successive cue onsets varied randomly between 5 and 5.5 sec as in the memorization task. The second task also had an easy and difficult version, each incorporating 48 stimuli in separate blocks. The accuracy and speed with which visual and auditory cues could be discriminated in these simple tasks were contrasted with discrimination performance during word list memorization.

### 2.4 Electroencephalography (EEG) recording and analysis

EEG was recorded from 32 scalp sites with sintered silver/silver-chloride electrodes embedded in an elastic cap. Electrodes were positioned according to an equidistant montage (www.easycap.de/easycap/e/electrodes/13_M10.htm). Vertical and horizontal eye movements were recorded bipolarly from electrodes placed above and below the right eye and on the outer canthus of each eye. A midfrontal site (corresponding to Fz in the 10/20 system) was used as the online reference. Impedances were kept below 5 kΩ. Online, signals were amplified, band-pass filtered between .01 and 35 Hz (3 dB roll-off), and digitized at a rate of 500 Hz (12-bit resolution). Offline, the data were digitally filtered between .05 and 20 Hz with a 96 dB roll-off, zero phase shift filter and algebraically re-referenced to linked mastoids. The online midfrontal site was re-instated and used as a scalp site of interest. Signals were downsampled to 100 Hz to assess cue-related activity and to 125 Hz to assess word-related activity.

The primary interest was in encoding-related activity elicited by cues. However, for completeness, we also computed encoding-related activity elicited by words. Activity elicited by cues and words was analyzed separately to allow each to be aligned to the time period immediately before each event (Galli et al., 2011; Gruber and Otten, 2010; Otten et al., 2006, 2010). This approach assesses whether words elicit encoding-related activity above and beyond any encoding-related activity elicited by cues. EEG epochs of 2560 and 2048 msec duration were extracted from the continuous record surrounding cues and words, respectively, each starting 100 msec before their onset. The slight differences in epoch length reflected the periods of time in which encoding-related effects were expected. Event-related potentials (ERPs) were generated for each participant and electrode site, separately for cues in each modality and discrimination difficulty condition. Blink artifacts were minimized with a linear regression procedure (Rugg et al., 1997) and trials containing non-blink eye movements, drifts (±50 μV), amplifier saturation, or muscle artifacts were excluded from the averaging process.

ERP waveforms for easy and difficult trials in both modalities were contrasted depending on whether the word on those trials was later recalled or forgotten (Sanquist et al., 1980). The average numbers of trials containing recalled and forgotten words were respectively 51 and 36, with negligible differences across experimental conditions. For cue-related activity, waveforms were quantified by measuring mean amplitudes in the 300–1000, 1000–2000, and 2000–2400 msec latency intervals following cue onset. Encoding-related activity elicited by words was quantified by measuring mean amplitudes in the 700–1200 and 1200–1900 msec intervals following word onset. The Results section provides a justification for these intervals. The analyses were performed across 26 electrode sites to assess scalp distribution differences across anterior and posterior sites (cf. Galli et al., 2011, 2012). The analyses of variance (ANOVAs) incorporated factors of scalp location (anterior/posterior) and electrode site (13 locations) in addition to the experimental factors of subsequent memory (recalled/forgotten), discrimination difficulty (easy/difficult) and stimulus modality (visual/auditory). Greenhouse–Geisser corrections were used for violations of sphericity (Keselman and Rogan, 1980). Lower order interactions were not considered in the presence of higher order interactions and only effects involving subsequent memory are reported.

## 3 Results

### 3.1 Recall performance

On average, 55.9% (SD = 15.3) of visual words were recalled following easy cue discriminations and 55.6% (SD = 14.1) following difficult cue discriminations. For auditory words, these values were respectively 57.9% (SD = 13.1) and 56.2% (SD = 11.9). A repeated measures ANOVA with factors of discrimination difficulty (easy/difficult) and stimulus modality (visual/auditory) did not suggest significant differences in recall (*p* > .368).

**Fig. 2:**
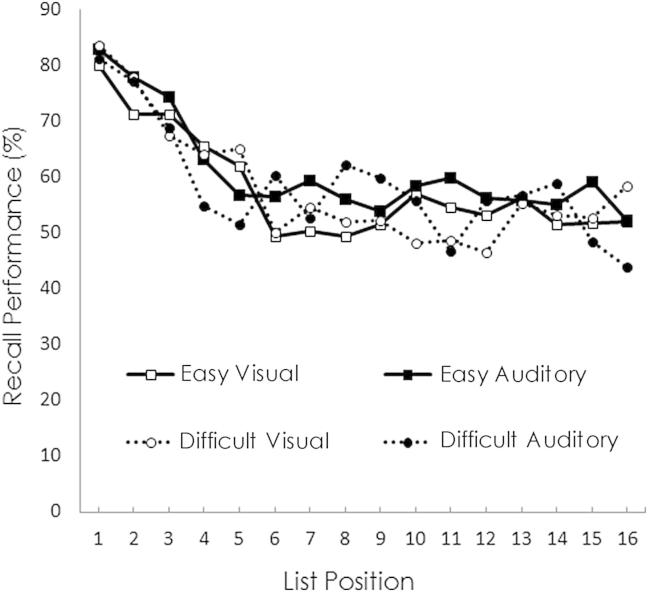
Serial position curves showing the percentages of recalled words in each of the 16 positions in the word lists. A primacy effect is evident for the first four positions. Recall performance for visual and auditory words in the easy and difficult cue discrimination conditions is virtually identical.

Fig. 2 shows the number of visual and auditory words recalled from each of the 16 positions in the easy and difficult discrimination lists. When the factor of list position was added to the ANOVA described above, a significant main effect of position emerged [Greenhouse–Geisser corrected *F*(7.04, 189.95) = 16.44, *p* < .001]. Confirming the visual impression of a primacy effect, pairwise comparisons on consecutive list positions indicated that recall was enhanced for words in the first four positions (*p* < .014; other *p* > .105). The ANOVA also showed a significant interaction between list position and stimulus modality [*F*(10.35, 279.40) = 1.99, *p* = .032]. This appeared to reflect the slightly higher recall of auditory than visual words from middle portions of the lists.

### 3.2 Cue discrimination performance

#### 3.2.1 Memorization task

**Fig. 3:**
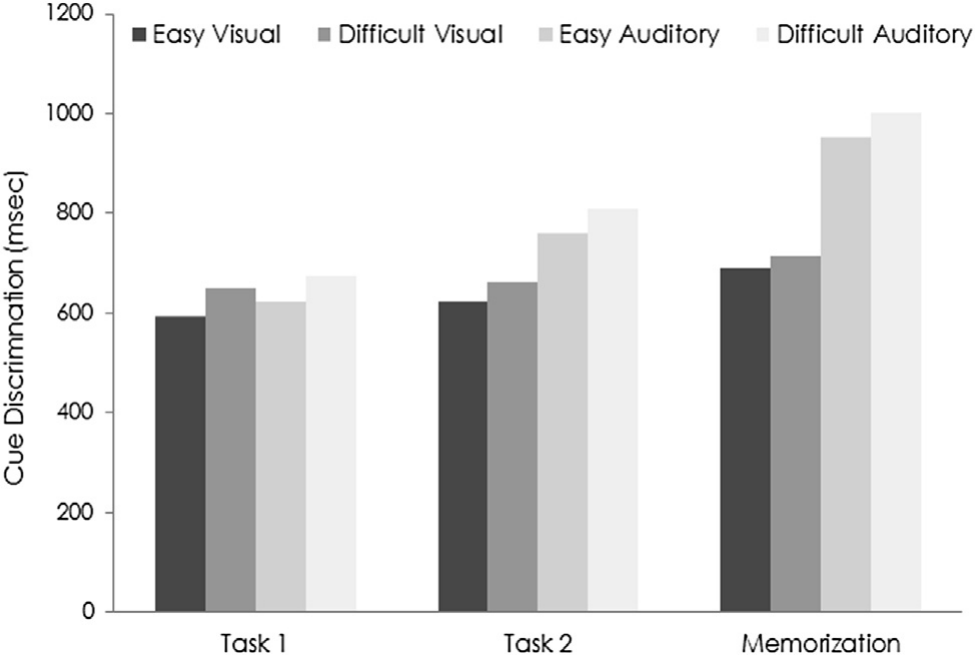
Response times associated with easy and difficult perceptual discriminations. The speed with which visual gratings and auditory tones were discriminated during the memorization task is shown on the right. On the left is discrimination performance during the simpler discrimination tasks. Task 1 refers to discriminations when intermixed gratings and tones were presented in isolation. Task 2 refers to discriminations when gratings and tones were presented in the same experimental sequence as used during memorization, except that neutral letter strings were used rather than words. In all three tasks, responses were slower when discriminations were difficult rather than easy. Regardless of difficulty, the time taken to discriminate increased from Task 1 to Task 2 to the memorization task. Response times were slower for auditory discriminations, except when such discriminations were made in relative isolation (Task 1).

During list learning, responses to prestimulus cues were more accurate and faster in the easy than difficult discrimination conditions (respectively 88.0% *vs* 83.7% and 822 *vs* 858 msec; Fig. 3). It also took on average less time to respond to visual than auditory cues (702 *vs* 978 msec). A repeated measures ANOVA on accuracy rates showed a main effect of discrimination difficulty [*F*(1, 27) = 8.76, *p* = .006]. This effect was also significant in the ANOVA on response times [*F*(1, 27) = 13.66, *p* = .001], along with a main effect of stimulus modality [*F*(1, 27) = 51.05, *p* < .001]. The interaction between input modality and discrimination difficulty was not significant for either accuracy or response time (*p* > .146).

Next, we assessed whether the time taken to discriminate prestimulus cues affected later memory performance. To this end, response times for the cue discriminations were sorted according to whether the word that followed the cue was later recalled or forgotten. In the easy condition, discrimination times preceding remembered and forgotten words were respectively 696 versus 701 msec for visual trials and 941 versus 983 msec for auditory trials. In the difficult condition, the corresponding times were 811 versus 736 msec for remembered and forgotten visual trials and 797 versus 1040 msec for remembered and forgotten auditory trials. These times were submitted to repeated measures ANOVA with factors of discrimination difficulty (easy/difficult), stimulus modality (visual/auditory), and subsequent memory (recalled/forgotten). This ANOVA gave rise to a significant three-way interaction [*F*(1, 27) = 27.44, *p* < .001]. Separate ANOVAs in each difficulty condition to understand the nature of this interaction resulted in significant two-way interactions between stimulus modality and subsequent memory for the easy [*F*(1, 27) = 5.07, *p* = .033] and difficult [*F*(1, 27) = 40.04, *p* < .001] conditions. In the easy condition, a main effect of subsequent memory occurred for auditory [*t*(27) = −2.17, *p* = .039] but not visual (*p* > .611) trials. In the difficult condition, main effects of subsequent memory were observed for auditory [*t*(27) = −7.40, *p* < .001] as well as visual [*t*(27) = 2.94, *p* = .007] trials. These analyses indicate that the speed with which cue decisions were made affected the likelihood of successful encoding, especially for auditory trials in the difficult discrimination condition. Faster cue responses were associated with better recall of auditory items, whereas this pattern was reversed for visual items.

#### 3.2.2 Simple discrimination tasks

To help understand the influence of cue discrimination difficulty on encoding-related brain activity, we administered two simple perceptual discrimination tasks on the stimuli used as prestimulus cues during list learning. Task 1 involved the discrimination of gratings and tones presented in relative isolation. Task 2 involved the discrimination of gratings and tones presented in the same experimental sequence as used during list learning, except that neutral stimuli rather than words were employed. Fig. 3 shows the speed of cue discriminations during Task 1, Task 2, and list learning. A repeated measures ANOVA with factors of discrimination difficulty (easy/difficult), modality (visual/auditory), and task (Task 1/Task 2/Memorization) revealed a main effect of discrimination difficulty [*F*(1, 27) = 19.05, *p* < .001]. This reflected the fact that response times were faster for easy discriminations. A main effect of task [*F*(1.3, 35.2) = 61.64, *p* < .001] indicated a gradual increase in response times from Task 1 to Task 2 [*t*(27) = 5.88, *p* < .001], and from Task 2 to Memorization [*t*(27) = 8.06, *p* < .001]. The interaction between task and modality was also significant [*F*(1.7, 46.3) = 45.30, *p* < .001]. Auditory discriminations were slower than visual discriminations during Task 2 and Memorization [*t*(27) = 5.70 and 7.14, respectively, both *p* < .001], but not during Task 1 (*p* = .228). Discrimination accuracy was not considered because it was close to ceiling during the simple discrimination tasks.

### 3.3 Encoding-related brain activity before word onset

**Fig. 4:**
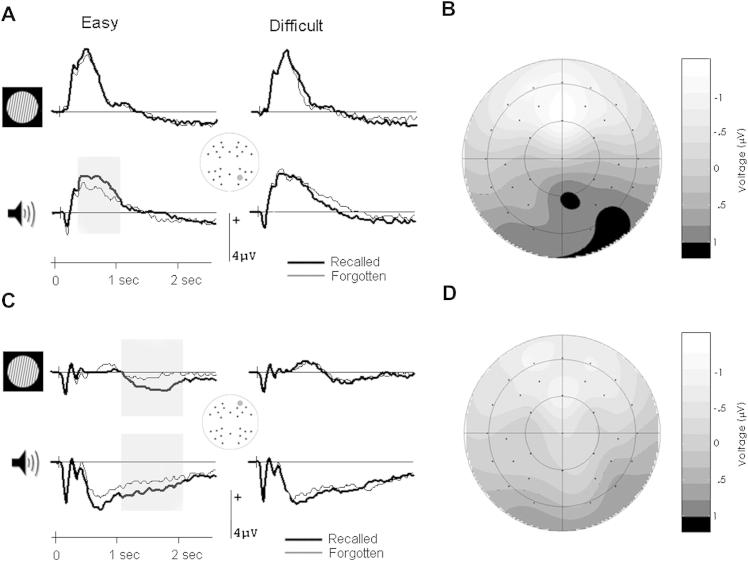
Electrical brain activity before word onset as a function of input modality and cue discrimination difficulty. (A) Group averaged ERP waveforms from a parietal scalp site (site 26 from montage 10, www.easycap.de/easycap/e/electrodes/13_M10.htm) in each experimental condition. Activity differed according to later recall performance shortly after the onset of auditory cues in the easy discrimination condition. (B) Voltage spline map of the ERP difference shown in (A). The map shows the activity for auditory trials in the easy discrimination condition in the 300–1000 msec interval after cue onset, scaled to the maximum and minimum in this condition. Shortly after the presentation of the cue, encoding-related activity is largest over posterior scalp sites, becoming smaller toward the front. (C) Waveforms from a left frontal scalp site (site 36 from montage 10). Brain activity before visual and auditory words show a sustained negative-going modulation when the words were later recalled. This is only evident when the cues preceding the words were easy to discriminate. (D) Voltage spline map of the ERP difference shown in (C). The map visualizes the distribution across the scalp of the difference between recalled and forgotten trials in the easy discrimination condition. The map shows activity averaged across visual and auditory trials in the 1000–2000 msec interval following cue onset, scaled to the same minimum and maximum as the map shown in (B). Encoding-related activity is largest over anterior scalp sites during this period of time. For graphical purposes, waveforms in (A) and (C) were low-pass filtered at 19.4 Hz.

Fig. 4 shows the group averaged ERPs elicited by the prestimulus cues, separated as a function of whether the following word was later recalled or forgotten. Encoding-related differences are visible prior to visual and auditory words in the easy but not difficult cue discrimination condition. Shortly after cue onset, waveforms at posterior sites differed according to later memory performance. This effect was particularly evident for auditory cues and took the form of a more positive-going waveform preceding words that were later remembered (Fig. 4A and B). This difference was quantified by measuring mean amplitudes in the 300–1000 msec latency interval, which captured the positive deflection in the group average. The ANOVA gave rise to significant interactions between discrimination difficulty, subsequent memory, modality and scalp location [*F*(1, 27) = 4.93, *p* = .035], and between discrimination difficulty, subsequent memory, scalp location and electrode site [*F*(5.0, 135.2) = 2.30, *p* = .048]. These interactions were decomposed with separate ANOVAs in each discrimination condition in line with the experimental focus. The interactions between subsequent memory, modality and scalp location, and between subsequent memory, scalp location and electrode site were only significant in the easy discrimination condition [respectively *F*(1, 27) = 6.93, *p* = .014 and *F*(4.2, 113.6) = 4.57, *p* = .002]. In this condition, ERP waveforms were more positive-going for auditory cues on posterior [*F*(1, 27) = 11.15, *p* = .002] but not anterior (*p* = .060) scalp locations when the following word was later recalled. Encoding-related activity did not emerge at any scalp location for visual cues (*p* > .265) or in the difficult discrimination condition (*p* > .373).

At a later point in time, encoding-related activity elicited by cues involving an easy discrimination was evident in both modalities in the form of a sustained negative-going deflection at anterior scalp sites (Fig. 4C and D). This effect is already apparent during the posterior deflection discussed above, but is largest in the middle of the cue-word interval, diminishing in size shortly before word onset. The effect was quantified by measuring mean amplitude values in the 1000–2000 msec interval to avoid overlap with the earlier quantification and to concentrate on the middle of the cue-word interval (cf. Otten et al., 2010). A separate measure was taken at the end of the interval (2000–2400 msec) to establish the reliability of activity just before word onset.

The ANOVA on the data from the 1000–2000 msec interval gave rise to a significant interaction between discrimination difficulty, subsequent memory and scalp location [*F*(1, 27) = 6.82, *p* = .015], which was further modulated by electrode site [*F*(5.2, 140.4) = 3.03, *p* = .011]. Separate analyses in each discrimination difficulty condition revealed an interaction between subsequent memory and scalp location for the easy condition [*F*(1, 27) = 11.73, *p* = .002]. This interaction reflected a negative-going subsequent memory effect at anterior [*F*(1, 27) = 5.32, *p* = .029] but not posterior (*p* = .482) locations. Visual and auditory cues involving a difficult discrimination did again not elicit significant encoding-related effects (*p* > .216). No significant effects emerged in proximity of word onset for either difficulty condition (*p* > .116).

### 3.4 Encoding-related activity after word onset

**Fig. 5:**
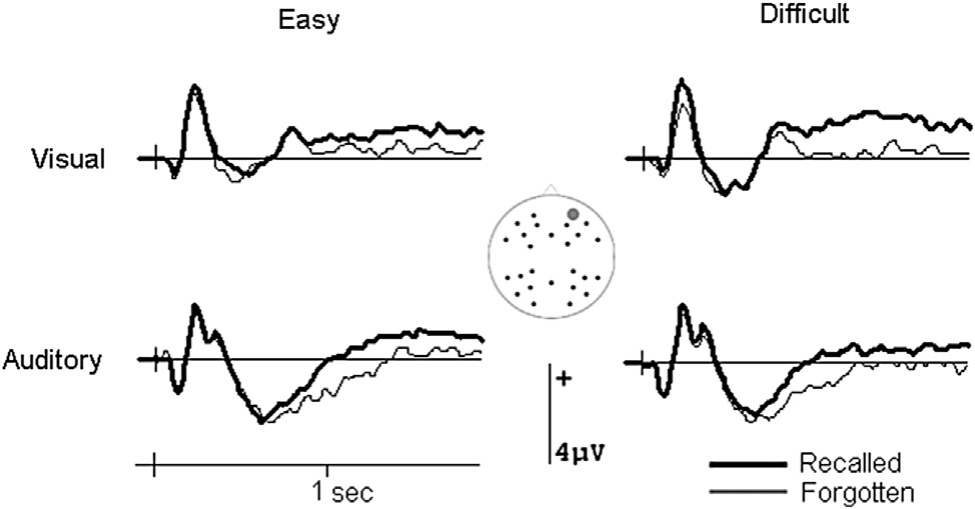
Encoding-related activity after word onset. Group averaged ERP waveforms from a left frontal scalp site (site 36 from montage 10, www.easycap.de/easycap/e/electrodes/13_M10.htm). Words elicited a positive-going subsequent memory effect over frontal scalp sites that did not differ as a function of discrimination difficulty or modality. Waveforms were low-pass filtered at 19.4 Hz for display purposes.

As typically observed (Friedman and Johnson, 2000), words that were later remembered elicited more positive-going waveforms over frontal scalp sites than words that were later forgotten (Fig. 5). Encoding-related activity elicited by words was quantified by measuring mean amplitudes in the 700–1200 and 1200–1900 msec intervals. These intervals were similar to those used to quantify post-stimulus subsequent memory effects in previous investigations (e.g., Galli et al., 2011; Otten et al., 2006, 2010) and captured the effects in the group averaged waveforms for all relevant conditions. The ANOVA revealed a significant interaction between subsequent memory and scalp location in both latency intervals [respectively *F*(1, 27) = 7.04 and 9.13, *p* = .013 and .005]. Subsequent memory effects were largest over anterior scalp sites, but significant at both anterior locations [*F*(1, 27) = 16.83 and 18.91 for the two intervals, both *p* < .001] and posterior locations [*F*(1, 27) = 10.49 and 8.13, respectively, *p* = .003 and .008]. No interactions involving modality or difficulty emerged (*p* > .117).

## 4 Discussion

The findings indicate that encoding-related activity before an event is sensitive to the degree to which processing resources are available. Electrical brain activity elicited by a cue presented just before word onset predicted later recall of the word, but only in a low demand situation when a concurrent task was easy to perform. Participants were asked to memorize short lists of words while making perceptual discriminations on cues that preceded the words. Discrimination difficulty was manipulated across lists by making the cues more or less similar to one another. The performance data show that cue discriminations were indeed faster and more accurate in the easy condition. The lower demands in this condition may have left sufficient opportunity to also engage brain activity that affects the encoding of the upcoming word. Accordingly, activity before word onset predicted later memory of the word. In the difficult discrimination condition, the need to discern the fine perceptual details of the cues likely left too few resources to also set up encoding-related activity in the available amount of time.

The influence of cue discrimination difficulty on encoding-related activity before an event suggests that the activity is limited in capacity and dependent on other ongoing processes. This observation narrows down the functional role that can be assigned to such activity. The findings may be more compatible with an interpretation of the prestimulus activity observed here as an active preparatory process (Otten et al., 2010) or an increase in general attention (Park and Rugg, 2010) rather than a naturally occurring state that is especially conducive to effective encoding (Meeter et al., 2004; Yoo et al., 2012). A caveat in this respect is that it is not possible to discern the precise nature of the processing resources that govern encoding-related activity on the basis of the current data alone. This is not a criticism of our study per se but the dual task paradigm more generally. The perceptual discrimination task that we used involves a number of functional processes, including perception, attention, working memory, decision making, and action control. Any of these processes could have interfered with the concurrent task of setting up encoding-related activity. Regardless, however, the current findings unequivocally demonstrate that engaging encoding-related activity before an event is not automatic but dependent on the availability of sufficient resources. This may explain why anticipatory influences on memory are observed in some situations and individuals but not others (e.g., Galli et al., 2011).

The main type of prestimulus activity observed in the present experiment was a negative deflection over anterior scalp sites. This deflection strongly resembles the activity repeatedly seen in semantic processing tasks (Otten et al., 2006, 2010; Padovani et al., 2011), including a recent investigation with experimental procedures similar to those employed here (Galli et al., 2012). Because the frontal negative deflection has thus far only been seen when an item's semantic and associative features are emphasized, this deflection is thought to reflect the adoption of mechanisms involved in the semantic processing of a stimulus ahead of stimulus presentation (Galli et al., 2012; Otten et al., 2006, 2010). Engaging such mechanisms early may enable the formation of a more elaborate and richer memory representation, which will be easier to retrieve later on (Craik and Lockhart, 1972). On this account, the difficult cue discrimination condition may have interfered with the engagement of semantic preparatory processes. The cue discrimination may have taken away attentional resources, a precursor for semantic processes. The fact that memory was affected by the time taken to discriminate the cue on individual trials supports this hypothesis. The amount of time allocated to the cue discrimination process leaves more or less opportunity to also set up encoding-related preparatory processes, affecting likelihood of later recall. The timing of encoding-related brain activity observed here is also consistent with the involvement of a preparatory process. The activity started around 1 sec after cue onset and ended just before word onset, similar to what has been observed previously when the input modalities of the cue and word are kept constant (Otten et al., 2010). The relatively late onset of the effect points to a preparatory process engaged in anticipation of the upcoming event rather than a cue-specific process.

Interestingly, we observed an additional prestimulus effect for auditory words. While the negative frontal effect occurred prior to visual and auditory words, a more posteriorly distributed effect was observed for auditory words in the easy cue discrimination condition. Activity shortly after the onset of auditory cues was more positive when the following word was later recalled. This effect was maximal over posterior scalp sites, suggesting a contribution of the P300 family of components (Donchin and Coles, 1988). Given the suggested role of the P300 in context updating and working memory, this might not seem surprising. The information about the upcoming input modality delivered by the cues is highly relevant and the better this information is processed, the more effective preparation might be. However, there seems little reason to assume why this would only be relevant for words presented in the auditory modality. We have previously noted that auditory words are special in the learning of short word lists (Galli et al., 2012). The same conclusion is evident from the fact that faster cue discrimination times increased likelihood of recall for auditory words, whereas recall was less likely for visual words. A special status of auditory information is also apparent from the simple discrimination tasks we gave participants. When visual gratings and auditory tones were presented in isolation, speed of discrimination was identical. This means that discriminations were not inherently easier for one or the other input modality. However, as soon as gratings and tones were presented in the same temporal sequence as used during memorization, discrimination times were slower for auditory decisions even though no words were presented. Although it is not clear how this translates to the positive prestimulus effect seen for auditory words, auditory processing must be especially sensitive to the temporal dynamics of the sequence in which stimuli are embedded. Importantly, the fact that this type of prestimulus activity was again only observed during the easy discrimination task emphasizes the importance of processing resources in the elicitation of prestimulus activity.

Brain activity after word onset was also predictive of subsequent memory performance. Words that were later remembered elicited more positive-going waveforms than words that were forgotten (Friedman and Johnson, 2000). In sharp contrast to what was seen for prestimulus activity, however, word-related activity did not differ as a function of discrimination difficulty or input modality. This indicates that encoding-related brain activity before a word is dissociable from activity thereafter, a finding that mimics earlier work (Galli et al., 2011; Otten et al., 2006). In the present case, this dissociation allows the strong conclusion that the difficulty manipulation successfully restricted the availability of processing resources to the time period before word onset and did not carry forward to the processing of the word itself.

A question worth exploring is whether the influence of processing resources on encoding-related activity before an event may relate to the manipulation of secondary task difficulty across blocks of trials. The use of a block design raises the possibility that sustained, state-related effects contributed to the findings. For three reasons, this does not seem likely. First, as mentioned above, encoding-related activity after word onset did not differ as a function of discrimination difficulty. Processing resources thus affected different periods of time within the same trial. Second, discrimination difficulty differentially affected visual and auditory cues, which were randomly intermixed. At least some effects of resource-availability must therefore be attributed to transient processes. Third, the time course of encoding-related activity before word onset is also inconsistent with state-related processes. Neural activity that is constant throughout a list should not emerge in item-related analyses or emerge very early after cue onset. Instead, encoding-related activity occurred in the middle of the cue-word interval in the present experiment. This time course is more consistent with a preparatory process that is engaged on each trial. In combination, the data suggest that preparatory processes act at the individual item level.

Even though neural activity before an event predicted the efficiency with which individual words were encoded into memory in the easy discrimination condition, overall recall performance did not differ as a function of cue discrimination difficulty. This contrasts with behavioral studies that typically show that dividing processing resources lowers memory performance (e.g., Fernandes and Moscovitch, 2000; Naveh-Benjamin et al., 2007). However, such studies manipulated resources after event onset and not before. Nonetheless, if difficult cue discriminations did indeed prevent the engagement of semantic preparatory processes, one might have expected recall to be poorer in that condition. This is not what we observed. The current study is certainly not unique in showing this pattern. Several studies show prestimulus activity that affects later memory performance in the absence of overall performance differences. For example, in a previous study on anticipatory processes and emotional memory encoding, we found that brain activity before unpleasant pictures predicts later memory performance in women, but not men (Galli et al., 2011). Overall memory performance, however, was identical across men and women. One explanation for the apparent discrepancy between the influence of preparation on encoding efficacy on individual trials and overall memory performance is that an influence of preparation during encoding may be compensated for at a later memory stage. On this account, any lack of preparation during encoding may result in a weaker representation that can nonetheless be retrieved because of compensatory processes engaged during consolidation, retrieval, or both. Preparatory processes during encoding are only one of many factors that determine whether an item will ultimately be remembered or forgotten.

In conclusion, we have demonstrated that encoding-related brain activity before an event varies as a function of the difficulty of a concurrent task. Prestimulus activity only seems to exert an influence on memory if sufficient processing resources are available for preparatory processes to unfold. This implies that the encoding of information into long-term memory can not only be enhanced by deploying attention once the information is presented, but also beforehand. It will be of interest to determine whether prestimulus activity that has been observed in other cognitive domains similarly depends on processing resources.
